# Image-based quantitative determination of DNA damage signal reveals a threshold for G2 checkpoint activation in response to ionizing radiation

**DOI:** 10.1186/2041-9414-1-10

**Published:** 2010-08-04

**Authors:** Aya Ishikawa, Motohiro Yamauchi, Keiji Suzuki, Shunichi Yamashita

**Affiliations:** 1 Atomic Bomb Disease Institute, Graduate School of Biomedical Sciences, Nagasaki University, 1-12-4 Sakamoto, Nagasaki 852-8523, Japan

## Abstract

**Background:**

Proteins involved in the DNA damage response accumulate as microscopically-visible nuclear foci on the chromatin flanking DNA double-strand breaks (DSBs). As growth of ionizing radiation (IR)-induced foci amplifies the ATM-dependent DNA damage signal, the formation of discrete foci plays a crucial role in cell cycle checkpoint activation, especially in cells exposed to lower doses of IR. However, there is no quantitative parameter for the foci which considers both the number and their size. Therefore, we have developed a novel parameter for DNA damage signal based on the image analysis of the foci and quantified the amount of the signal sufficient for G2 arrest.

**Results:**

The parameter that we have developed here was designated as SOID. SOID is an abbreviation of Sum Of Integrated Density, which represents the sum of fluorescence of each focus within one nucleus. The SOID was calculated for individual nucleus as the sum of (area (total pixel numbers) of each focus) x (mean fluorescence intensity per pixel of each focus). Therefore, the SOID accounts for the number, size, and fluorescence density of IR-induced foci, and the parameter reflects the flux of DNA damage signal much more accurately than foci number. Using very low doses of X-rays, we performed a "two-way" comparison of SOID of Ser139-phosphorylated histone H2AX foci between G2-arrested cells and mitosis-progressing cells, and between mitosis-progressing cells in the presence or absence of ATM or Chk1/2 inhibitor, both of which abrogate IR-induced G2/M checkpoint. The analysis revealed that there was a threshold of DNA damage signal for G2 arrest, which was around 4000~5000 SOID. G2 cells with < 4000 SOID were neglected by G2/M checkpoint, and thus, the cells could progress to mitosis. Chromosome analysis revealed that the checkpoint-neglected and mitosis-progressing cells had approximately two chromatid breaks on average, indicating that 4000~5000 SOID was equivalent to a few DNA double strand breaks.

**Conclusions:**

We developed a novel parameter for quantitative analysis of DNA damage signal, and we determined the threshold of DNA damage signal for IR-induced G2 arrest, which was represented by 4000~5000 SOID. The present study emphasizes that not only the foci number but also the size of the foci must be taken into consideration for the proper quantification of DNA damage signal.

## Background

Cell cycle checkpoint is the mechanism that secures integrity of the genome. It is activated by DNA damage caused by DNA damaging agents, such as ionizing radiation [1]. Activated checkpoints halt cell cycle progression or execute cell death. Three major cell cycle checkpoints induced by IR include G1 checkpoint preventing G1-S transition, intra-S checkpoint halting DNA replication, and G2/M checkpoint that inhibits G2 cells to enter mitosis [2]. The master regulator of the IR-induced cell cycle checkpoints is ataxia telangiectasia mutated (ATM) protein, a serine/threonine kinase which belongs to a phospho-inositide 3-kinase (PI3K)-related kinase family [3]. ATM protein form inactive dimers or higher-order multimers in unstressed cells, but it is activated through intermolecular autophosphorylation at Ser1981 and monomerization in response to alteration of chromatin structure induced by DNA double-strand breaks or other chromatin-perturbing treatments [4]. A recent proteomic study revealed that, in response to IR, ATM phosphorylates > 900 serine and/or threonine residues on > 700 proteins including factors involved in cell cycle checkpoints, such as Chk2 and p53 [5], and, thus, ATM transactivates DNA damage checkpoints. In G2/M checkpoint, ATM activates Chk2 through phosphorylation at Thr68 [6,7]. Then, activated Chk2 phosphorylates and negatively regulates CDC25C, which is the positive regulators for the activity of cdc2/cyclinB required for mitosis entry [8].

Recently, phosphorylated forms of such downstream factors have been treated as surrogate markers for DNA damage signaling. For example, several studies unraveled that histone H2AX, which is a subtype of histone H2A, and constitutes 2-25% of total H2A protein, was phosphorylated at Ser139 by ATM in response to DSBs. Phosphorylation of histone H2AX spans several mega base pairs of chromatin flanking DSBs [9-12], and thus, phosphorylated histone H2AX can be microscopically visible as nuclear foci by immunofluorescence staining using specific antibody recognizing phosphorylated forms of histone H2AX [13]. It is now generally considered that a focus of phosphorylated H2AX, also called as gamma-H2AX focus, represents a single DSB, because the number of foci per cell immediately after IR is very close to theoretically-estimated DSB number after given doses of IR [13]. Thus, phosphorylated H2AX foci are now widely used as an indicator for DSBs [14]. However, more recent studies also revealed that phosphorylated H2AX foci is not just an indicator for DSBs, but also a platform playing an essential role in DNA damage signaling. It was reported that a number of other proteins also form the colocalized foci with phosphorylated H2AX foci, whose colocalization was totally relied on H2AX phosphorylation. Such proteins include MDC1, 53BP1, RNF8, MRE11-Rad50-NBS1 complex [4,15-24]. Moreover, these foci-forming proteins are critical for accumulation of phosphorylated ATM at focal site, and therefore, they are considered to be involved in ATM-dependent DSB response [25-27]. Indeed, depletion of H2AX phosphorylation or colocalized factors negatively affects IR-induced checkpoint, especially, in cells exposed to lower doses of IR [17,21,24,28,29].

We previously demonstrated that persistent Ser1981-phosphorylated ATM foci grow in size after IR, and the foci size of the phosphorylated ATM is well correlated with phosphorylation levels of p53 at serine15, which is the direct target of ATM. It is indicated that foci growth could be an essential mechanism for amplifying the DNA damage signal for G1 checkpoint activation [30]. Otherwise, inappropriate DNA damage amplification fails in executing G1 arrest, as shown in AT and NBS cells [30]. While the DNA damage signal amplification is indispensable for G1 arrest, a role of amplification of DNA damage signal in G2 checkpoint activation remains to be determined.

In the present study, we developed a novel quantitative parameter for DNA damage signal. Because the number of foci is well correlated with the number of DSBs but the foci number might not be an appropriate index for the amount of DNA damage signal, our parameter integrates not only the number but also the size of IR-induced foci for proper quantification of DNA damage signal. The new parameter, SOID, represents the sum of fluorescence of each focus within one nucleus. The SOID was calculated for individual nucleus as the sum of (area (total pixel numbers) of each focus) x (mean fluorescence intensity per pixel of each focus), and it was expected to reflect the flux of DNA damage signal much more accurately than foci number. We performed a "two-way" comparison of SOID of Ser139-phosphorylated histone H2AX foci between G2-arrested cells and mitosis-progressing cells. The analysis revealed that there was a threshold of DNA damage signal for G2 arrest, which was around 4000~5000 SOID. Chromosome analysis revealed that the checkpoint-neglected mitosis-progressing cells had approximately two chromatid breaks on average, indicating that 4000~5000 SOID was equivalent to a few DNA double strand breaks.

## Results

### Quantification of DNA damage signal involved in G2/M checkpoint activation

To quantify DNA damage signal sufficient for G2 arrest we compared the amount of DNA damage signal detected in G2-arrested cells with that in mitosis-progressing cells after IR. For this purpose, we decided to use very low doses of X-rays (0.02~0.4 Gy). Because higher doses of X-rays, such as 1 Gy, completely arrested G2 cells in our normal human primary fibroblasts, it prevented examination of signal amount left in mitosis-progressing cells. For example, no mitotic cells were observed in 7440 cells analyzed 2 hr after 1 Gy. We also quantified DNA damage signal in mitosis-progressing cells exposed to IR in the presence of inhibitors for ATM or Chk1/2, which enabled G2 to mitosis progression irrespective of the amount of DNA damage signal. For quantification of DNA damage signal, we used the foci of Ser139-phosphorylated histone H2AX. The size of phosphorylated H2AX foci well correlated with that of Ser1981-phosphorylated ATM foci, and phosphoryated H2AX foci could be detectable in mitotic cells [31]. This was in contrast to the other DNA damage checkpoint factors like 53BP1, which were not detectable in mitosis [32].

**Figure 1:**
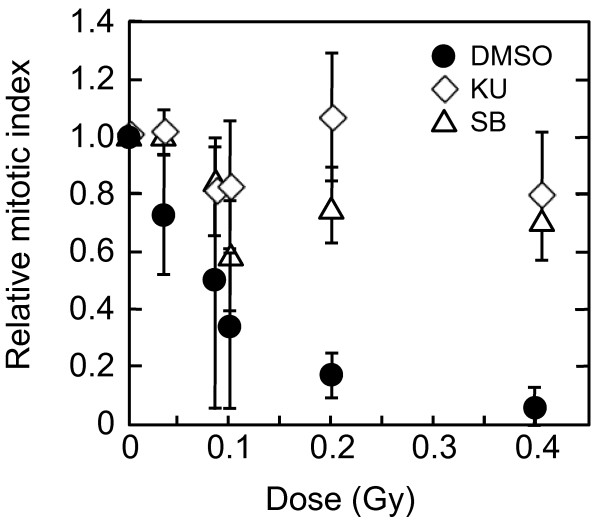
**G2 arrest and its inhibition induced by low doses of X-rays**. Exponentially-growing normal human primary fibroblasts were irradiated with 0, 0.02, 0.04, 0.08, 0.1, 0.2, and 0.4 Gy of X-rays, and two hours later, cells were fixed and subjected to immunofluorescence staining for Ser10-phosphorylated histone H3. KU55933 (10 μM), SB218078 (2.5 μM), or their solvent DMSO was administrated 30 min before IR. Mitotic cells were identified by strong fluorescence intensity of phosphorylated H3 signals. More than 5000 cells were scored at each dose and each treatment, and the results obtained from three independent experiments were pooled. Data indicate mean ± SD.

**Figure 2:**
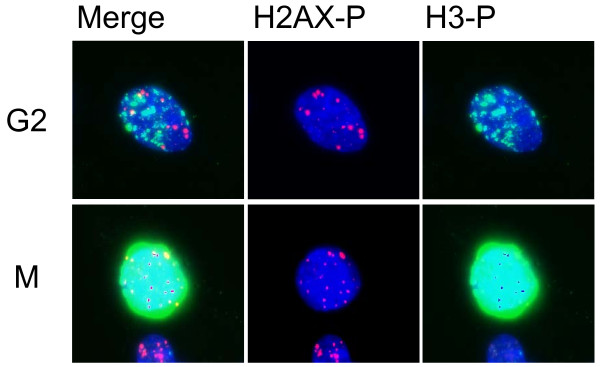
**Phosphorylated histone H2AX foci formed in G2 and mitotic cells**. Formation of phosphorylated H2AX foci and phosphorylation of histone H3 were examined in G2 cells and mitotic cells 2 hr after 0.4 Gy of X-rays. Note that G2 cells have weaker intensity and more rugged and discontinuous pattern of phosphorylated histone H3 staining compared to mitotic cells. In contrast, mitotic cells have foci with smaller size and weaker fluorescence intensity than G2 cells.

**Figure 3:**
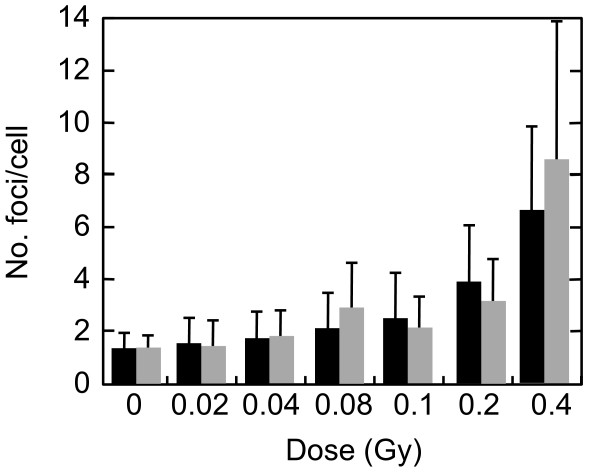
**Dose-dependent induction of foci in G2 and mitotic cells**. Exponentially-growing normal human primary fibroblasts were irradiated with 0, 0.02, 0.04, 0.08, 0.1, 0.2, and 0.4 Gy of X-rays, and two hours later, cells were fixed and subjected to immunofluorescence staining for Ser139-phosphorylated H2AX and Ser10-phosphorylated H3. Foci numbers of Ser139-phosphorylated histone H2AX in G2 cells and mitotic cells were counted. G2 cells (black bars) and mitotic cells (grey bars) were identified as described in Figure 2. Data indicate means ± SD.

First, we examined the mitotic index 2 hr after irradiation with 0.02, 0.04, 0.08, 0.1, 0.2, and 0.4 Gy of X-rays in the presence or absence of KU55933 (10 μM) or SB218078 (2.5 μM), which is well-established inhibitor for ATM and Chk1/2, respectively [33,34]. Mitotic cells were identified by immunofluorescence staining of Ser10-phosphorylated histone H3. The mitotic index was decreased dose-dependently in the absence of the inhibitors, indicating that G2 arrest was efficiently induced even by low doses of X-rays (Figure 1). We found that G2 arrest was largely dependent on ATM-dependent chk2 activation, as it was almost abrogated in the presence of KU55933 or SB218078 even after 0.4 Gy (Figure 1). Next, we compared the number of phosphorylated H2AX foci between G2 cells and mitotic cells 2 hr after X-irradiation. G2 cells were distinguished from mitotic cells by weaker intensity and more rugged and discontinuous pattern of phosphorylated histone H3 staining. Representative photos presented in Figure 2 showed that the number of foci in mitosis-progressing cells was not always less than that observed in G2 cells. However, we noted that the size of the foci was much smaller in mitosis-progressing cells. Dose-dependent induction of foci in G2 and mitotic cells, shown in Figure 3, also indicated that there was no apparent difference in the foci numbers between G2 cells and mitotic cells. For example, similar foci numbers were observed in G2 and mitotic cells exposed to 0.4 Gy of X-rays, whose dose clearly induced G2 arrest in substantial proportion of cells (Figure 1). Because the weaker fluorescence intensity was commonly observed in the foci of mitosis-progressing cells, it was indicated that the size of foci in addition to the foci number should be taken into consideration, when the amount of DNA damage signal was evaluated based upon the foci. Therefore, we invented a novel parameter, into which the foci number, the foci size, and fluorescence intensity of each focus were all integrated.

**Figure 4:**
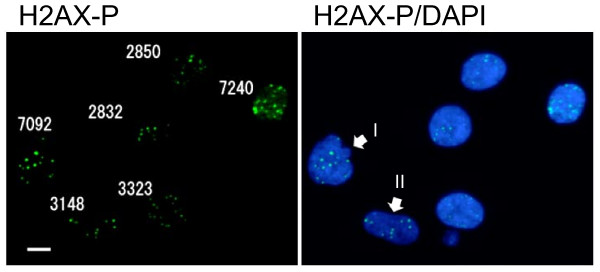
**Representative SOID measurements in G2 cells**. The SOID values were examined in cells 2 hours after exposure to 0.4 Gy of X-rays. The SOID was measured as described in Methods. Left panel: the SOID values in each nucleus. Right panel: white arrows indicate two nuclei with similar foci numbers but different the SOID values.

**Figure 5:**
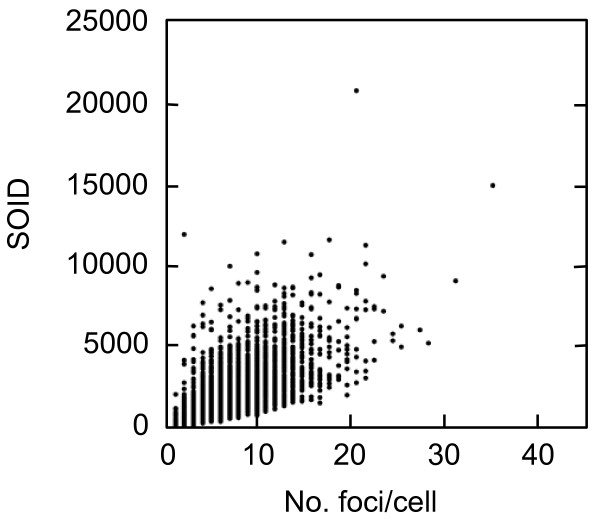
**Relationship between the number and the SOID of foci per cell**. Exponentially-growing normal human primary fibroblasts were irradiated with 0.4 Gy of X-rays, and two hours later, cells were fixed and subjected to immunofluorescence staining for Ser139-phosphorylated H2AX and Ser10-phosphorylated H3. The SOID values and the number of foci were examined in G2 cells. The SOID was measured as described in Methods. The SOID values and the corresponding foci numbers were plotted.

**Figure 6:**
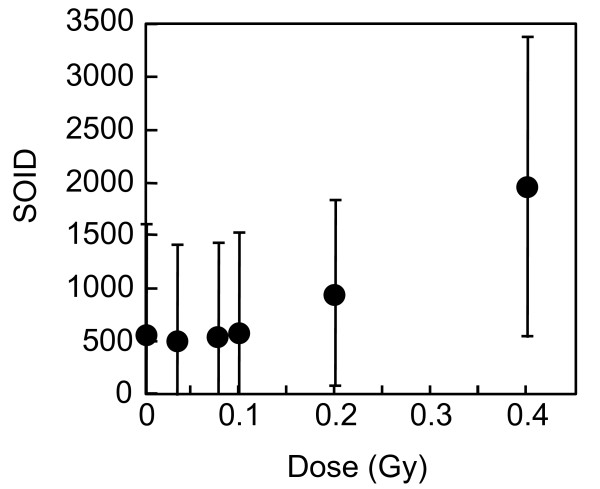
**Dose-dependent increase of the SOID in G2 cells**. Exponentially-growing normal human primary fibroblasts were irradiated with 0, 0.02, 0.04, 0.08, 0.1, 0.2, and 0.4 Gy of X-rays, and two hours later, cells were fixed and subjected to immunofluorescence staining for Ser139-phosphorylated H2AX and Ser10-phosphorylated H3. The SOID values were examined in G2 cells. The SOID was measured as described in Methods. Data indicate means ± SD.

The new parameter was designated as SOID, which represents the sum of fluorescence of each focus within one nucleus. The SOID was calculated for individual nucleus as the sum of (area (total pixel numbers) of each focus) x (mean fluorescence intensity per pixel of each focus). As shown in Figure 4, SOID values were calculated for each nucleus. For example, the numbers of foci in I and II nuclei are 12 and 9, respectively, whereas the SOID values are calculated as 7092 and 3148. The relationship between the SOID values and the numbers of foci was examined in cells exposed to 0.4 Gy of X-rays (Figure 5). We observed no close relation between the numbers and the SOID values by linear regression analysis (correlation coefficient R = 0.68), confirming that the foci numbers alone were insufficient for evaluating the amount of DNA damage signal. Then, dose-dependent increase in the SOID values is determined 2 hours after 0.4 Gy of X-rays (Figure 6). At this time point, the number of foci was approximately a half of that of the foci initially formed, according to DNA repair. Some of the foci became smaller, while the persisted foci tended to grow. Therefore, the amount of fluorescence of each focus was quite different. As a result, the SOID values showed large deviation, but we observed a tendency of dose-dependent increase above 0.1 Gy.

### Threshold of SOID for G2 arrest

In order to determine a threshold for G2 arrest, we performed a "two-way" comparison of the SOID. Namely, one is between G2-arrested cells and mitosis-progressing cells, and the other is between mitotic cells cultured after X-irradiation in the presence or absence of G2/M checkpoint inhibitors. Exponentially-growing normal human primary fibroblasts were irradiated with 0.4 Gy of X-rays and fixed at 2 hr after IR. The inhibitors or their solvent DMSO was administrated 30 min before IR until 30 minutes before the time of sample preparation. Because KU55933 by itself affected the foci formation of phosphorylated histone H2AX, it was washed out 30 min before fixation to recover size and fluorescence intensity of the foci.

**Figure 7:**
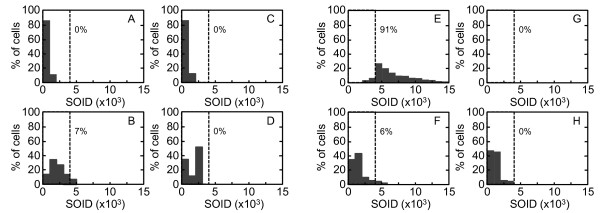
**Distribution of the SOID values in G2 and mitotic cells**. Exponentially-growing normal human primary fibroblasts were irradiated with 0.4 Gy of X-rays (A - D). Two hours after X-irradiation, cells were fixed and subjected to immunofluorescence staining for Ser139-phosphorylated H2AX and Ser10-phosphorylated H3. Cells were also exposed to 1.0 Gy of X-rays (E - H). They were fixed and subjected to immunofluorescence staining for Ser139-phosphorylated H2AX and Ser10-phosphorylated H3 at 2 hours (E and G), 6, 8 and 12 hours (F and H) later. The SOID values of phosphorylated H2AX foci in the control cells (A and C), 0.4 Gy-irradiated cells (B and D), and 1.0 Gy-irradiated cells (E - H) were measured. The SOID values obtained from 1.0 Gy-irradiated cells prepared at 6, 8 and 12 hours later were compiled in F and H. G2 cells (A, B, E and F) and mitotic cells (C, D, G and H) were identified as described in Figure 2. Dotted lines indicate 4000 SOID, and the numbers indicate the percentage of cells with the SOID above 4000.

**Figure 8:**
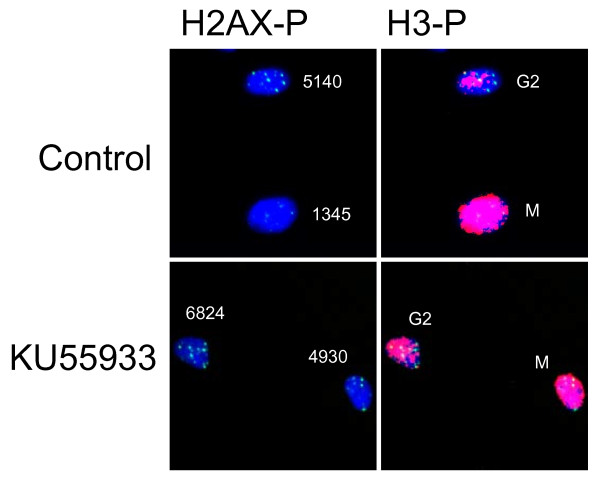
**Representative SOID measurement in G2 cells and mitotic cells in the presence of KU55933**. KU55933 (10 μM), or their solvent DMSO was treated from 30 min before IR. Two hours later, cells were fixed and subjected to immunofluorescence staining for Ser139-phosphorylated H2AX and Ser10-phosphorylated H3. KU55933 was washed out 30 minutes before fixation to recover the size and the fluorescence intensity of phosphorylated H2AX foci. Numbers indicate the SOID values in each nucleus.

**Figure 9:**
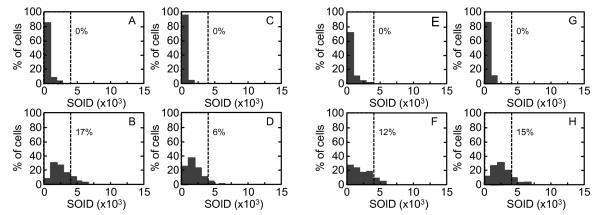
**Distribution of the SOID values in G2 and mitotic cells treated with ATM or CHK1/2 inhibitor**. Exponentially-growing normal human primary fibroblasts were irradiated with 0.4 Gy of X-rays in the presence of KU55933 (A - D) or SB218078 (E - H). Two hours later, cells were fixed and subjected to immunofluorescence staining for Ser139-phosphorylated H2AX and Ser10-phosphorylated H3. The SOID values of phosphorylated H2AX foci in the control cells (A, C, E and G) and 0.4 Gy-irradiated cells (B, D, F and H) were measured. G2 cells (A, B, E and F) and mitotic cells (C, D, G and H) were identified as described in Figure 2. Dotted lines indicate 4000 SOID, and the numbers indicate the percentage of cells with the SOID above 4000.

A clear difference in the SOID distribution was observed at 0.4 Gy. The SOID values spanned between 0 to 6000 in 0.4 Gy-irradiated G2 cells (Figure 7B), however, mitotic cells with > 4000 SOID were rarely observed after 0.4 Gy (Figure 7D). We found that 7% of cells showed the SOID over 4000 in G2 cells but not in mitosis-progressing cells. This result suggested that cells with > 4000 SOID were unable to enter mitosis. Therefore, we confirm this with cells exposed to 1.0 Gy of X-rays. As 1.0 Gy of X-rays induced G2 arrest, no mitotic cells were detected 2 hours after X-irradiation (Figure 7G). In G2 cells, we found the SOID values expanding for over 15000 (Figure 7E). Since we found cells released from G2 arrest after 6 hours, the samples were prepared at 6, 8 and 12 hours after X-irradiation. The compiled data showed that 6% of G2 cells had the SOID over 4000 (Figure 7F), whereas that of mitotic cells was below 4000 (Figure 7H). Then, the SOID was compared between G2 cells and mitosis-progressing cells exposed to 0.4 Gy of X-rays in the presence or absence of the inhibitors. The SOID value in mitosis-progressing cells is significantly lower than that of G2 cells, while they are not very different when ATM activity is inhibited (Figure 8). In cells treated with KU55933 or SB218078, the SOID value spanned 0 - 7000 and 0 - 8000 in mitosis-progressing cells, respectively (Figures 9D and 9H), and we confirmed the SOID distribution was not significantly varied between G2 and mitotic cells. Thus, it was confirmed that G2 cells with > 4000 SOID was restricted to progress into mitosis in the presence of G2/M checkpoint. While most of the mitosis-progressing cells showed SOID value not more than 3000, there were very few but some mitosis-progressing cells with more than 3000 SOID. Therefore, we concluded that the threshold of SOID value for G2 arrest was estimated to be between 4000~5000.

### Relationship between SOID value and the number of chromomatid breaks

**Figure 10:**
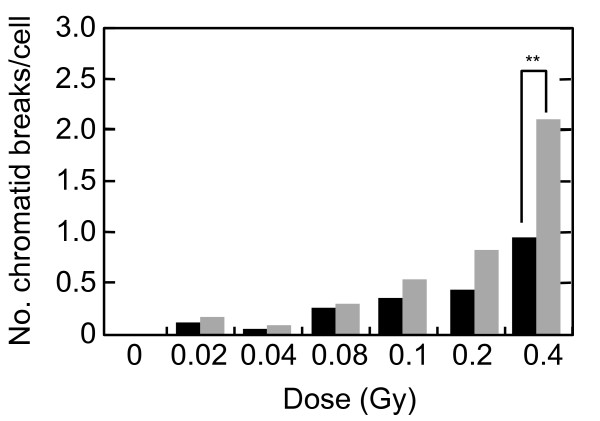
**Dose-dependent induction of chromatid breaks**. Exponentially-growing normal human primary fibroblasts were irradiated with 0, 0.02, 0.04, 0.08, 0.1, 0.2, and 0.4 Gy of X-rays, and two hours later, mitotic cells were harvested and chromosome samples were prepared. Colcemid (0.1 μg/ml) was treated from immediately after IR to 2 hr after IR in order to collect metaphase cells. KU55933 (10 μM) was treated from 30 min before IR to 1.5 hr after IR, and washed out, then media were replaced by fresh media containing DMSO and Colcemid. After the media replacement, cells were cultured for additional 30 min, followed by metaphase harvest. More than 50 metaphases were examined per point. Black bars: control cells, Grey bars: KU55933-treated cells. ** indicates significant difference at p < 0.01.

As the threshold of SOID value for G2 arrest was estimated to be between 4000~5000, we then asked what is the cytological damage corresponding to 4000~5000 SOID. We analyzed chromatid breaks in mitosis-progressing cells 2 hr after 0.02-0.4 Gy of X-rays (Figure 10). Colcemid (0.1 μg/ml) was treated from immediately after IR to 2 hr after IR to collect metaphase cells. Here, we again used KU55933 to inhibit G2/M checkpoint. To make experimental setting consistent with the SOID analysis, KU55933 was washed out 30 min before metaphase harvest. We found the induction of chromatid breaks with doses ≥ 0.02 Gy, but the frequency was not affected by KU55933 treatment in X-irradiated population with up to 0.08 Gy. This was in agreement with the result that 0.02-0.08 Gy of X-rays induce G2 arrest only a fraction of cells, if any (Figure 1). At higher doses the difference became more evident. With 0.4 Gy of X-rays, approximately one chromatid break per cell was observed in mitosis-progressing cells, whereas it was significantly increased by KU55933-treatment (p < 0.01). Average number of chromatid breaks per cell were 0.96 and 2.08 in the control and KU55933-treated population, respectively. As approximately 45% of metaphases showed chromatid breaks (Table 1), the number of chromatid breaks in cells with chromosome aberrations was estimated as around two. Thus, it was indicated that 4000~5000 SOID was equivalent to approximately two chromatid breaks, which correspond to a few DNA double strand breaks.

**Table 1:** chromatid breaks induced by various doses of x-rays

Treatment	No. metaphases analyzed	No. metaphases with aberrations	No. of chromatid breaks
Control (+DMSO)			
0 Gy	55	1	1
0.02 Gy	59	7	8
0.04 Gy	61	3	3
0.08 Gy	75	11	16
0.1 Gy	70	15	21
0.2 Gy	50	14	18
0.4 Gy	55	25	53
			
+KU55933			
0 Gy	54	0	0
0.02 Gy	56	9	9
0.04 Gy	64	5	5
0.08 Gy	61	11	15
0.1 Gy	53	19	25
0.2 Gy	50	31	42
0.4 Gy	51	42	106

Cells were treated with KU55933 (10 μM) 30 minutes before irradiation until 30 minutes before metaphase harvest. Immediately after irradiation, colcemid (0.1 μM) was added to the medium, and cells were incubated for 2 hours. Then, metaphases were collected and chromosome samples were prepared as described in Methods.

## Discussion

Here we developed a novel parameter for quantifying DNA damage signal considering both the number and the size of the foci induced by IR. The new parameter, SOID, reflected an integrated amount of DNA damage signal in a single cell. We previously demonstrated that Ser15-phosphorylation level of p53 depends on focus size of Ser1981-phosphorylated ATM, and a single persistent phosphorylated ATM focus can deposit and emit DNA damage signal sufficient for G1 checkpoint induction through focus growth [30]. The finding indicates that not only the foci number, but also the foci size must be taken into consideration when DNA damage signal of foci is quantified. Indeed, we failed to observe significant difference in the number of phosphorylated H2AX foci between G2-arrested cells and mitosis-progressing cells after 0.4 Gy, while the dose induced apparent G2 arrest (Figure 3). In contrast, the SOID values visualized the difference between G2-arrested cells and mitosis-progressing cells. Thus, SOID could be a valuable parameter to qualify the amount of DNA damage signal required for G2/M checkpoint activation. As shown in Figure 4, it was quite evident that there was marked variations in the size and fluorescence intensity in each focus. Although the reason for the difference is currently unknown, one possible explanation could be that a focus with smaller size and weaker intensity may represents a residual signal of a DSB that is rejoined just before sample fixation. In any case, such possibility could also be the reason why the similar number of foci gives different SOID value between G2-arrested cells and mitosis-progressing cells. Thus, it can be concluded that the SOID is the better indicator for the quantity of DNA damage signals than the foci number alone.

A two-way comparison of the SOID between G2-arrested cells and mitosis-progressing cells, and between mitosis-progressing cells in the presence or absence of ATM or Chk1/2 inhibitor revealed that there was a threshold of SOID for G2 arrest, which is about 4000~5000. Our results demonstrated that most G2 cells with < 3000 SOID can evade G2/M checkpoint, however, there still be some few mitosis-progressing cells more than 3000 SOID. Therefore, it is more appropriate to conclude that the threshold of SOID for G2 arrest is about 4000~5000. Chromosome analysis revealed that such checkpoint-neglected cells progress to mitosis harbored ~2 chromatid breaks/cell. According to the previous estimation, in which one premature chromosome condensation (PCC) break is equated to 3~6 DSBs, 4000~5000 SOID could correspond to similar amount of DSBs [35]. In fact, foci number in Figure 3 was 7~9, which was comparable to the estimation. In contrast, inhibition of G2/M checkpoint by KU55933 or SB218078 allowed cells with ≥ 4000 SOID to enter mitosis (Figures 9). Inhibition of G2/M checkpoint by KU55933 also increased the number of chromatid breaks/cell, which was most pronounced after 0.4 Gy. These results indicate that the SOID value ≥ 4000 is biologically relevant. Cells with such amount of DNA damage signal of IR-induced foci elicit G2/M checkpoint, thereby minimizing the frequency of chromosome aberration in mitosis-progressing cells.

Previously, Deckbar et al. reported that G2/M checkpoint was imperfect, and its release occurred at a point when ~3.5 PCC breaks and 10~20 phosphorylated H2AX foci left. Based on the above estimation, they correspond to 10~20 DSBs remained [35]. We found that their threshold was clearly higher than that obtained in the present study. Although the reason for this discrepancy is not clear, a couple of points can be discussed. One of which is the size of the foci. We observed that there was an inverse relationship between the size and the number of foci. In fact, the size of the foci in cells with 10~20 foci was relatively small. Therefore, it seemed likely that the integrated DNA damage signal might be lower than the threshold, even the number of foci was 10~20. The second point could be the procedure used for counting the foci number. In the previous study, foci numbers in CENP-F-positive G2 cells exposed to 1 Gy of X-rays were counted. The number of the initial foci could be higher, and foci number was not determined in mitosis released from G2 arrest. In our examination, we counted foci numbers in phospho-H3-positive mitotic cells, predominantly in prophases. Therefore, we could determine the exact number of foci in cells passed through G2 arrest. Although these might or might not be the primary reason for the discrepancy, these observations again strengthened our claim that not only the number but also the size of the foci must be considered in order to quantify the amount of DNA damage signal based on the foci.

Since the foci of phosphorylated histone H2AX were proved to be the most suitable and trustable surrogate marker for DSBs, several procedures have been developed to quantitate the amount of foci [14]. Once a reliable antibody against phophorylated H2AX foci was established, image-based assay was introduced to count the number of foci [36]. However, as described above, these assays were to count the number of foci and they were unable to measure the size of foci. Subsequently, flow-cytometry was introduced for automatic quantification of DNA damage signal based on total fluorescence obtained by immunofluorescnce assay [37,38], however, the assay could not account for the number of foci. Our current technique could unite these two procedures, which made the quantification of both the foci number and the size possible. The SOID value could be a novel parameter to evaluate DNA damage signal essential for genome integrity maintenance.

## Conclusions

We developed a novel parameter for quantitative analysis of DNA damage signal, and we determined the threshold of DNA damage signal for IR-induced G2 arrest, which was represented by SOID 4000~5000. The present study emphasized that not only the foci number but also the size of the foci must be taken into consideration for the proper quantification of DNA damage signal.

## Methods

### Cell culture and irradiation

Low passage (4-9) normal human diploid primary fibroblasts were cultured in minimal essential Eagle's media (MEM) containing 10% fetal bovine serum (Thermo Fisher Scientific, USA) [30]. One to 4 × 10^4^cells were seeded onto 22 x 22 mm coverslips in 35 mm dishes. Two days later, cells were irradiated with X-rays from X-ray generator (ISOVOLT TITAN 320, GE, USA) at 200 kV and 15 mA with a 0.5 mm copper filter at a dose rate of 0.2082 Gy/min. ATM inhibitor, KU55933 (Calbiochem, USA) was dissolved in DMSO to prepare 20 mM stock solution, and was treated at a final concentration of 10 μM. The KU55933 was treated from 30 min before X-ray-irradiation, and was washed out 30 min before fixation to recover size and fluorescence intensity of phosphorylated H2AX foci. Chk1/2 inhibitor, SB218078 (Calbiochem, USA) was dissolved in DMSO to prepare 2.5 mM stock solution, and was treated at a final concentration of 2.5 μM. The SB218078 was treated from 30 min before X-ray-irradiation to the time of fixation.

### Immunofluorescence staining

Cells were once washed with 1 x PBS^-^, and fixed with 4% formaldehyde in 1 x PBS^- ^for 10 min, then permeabilized with 0.5% Triton X-100 in 1 x PBS^- ^for 5 min. After permeabilization, the primary antibodies were applied for 2 hr in a 37°C humidified CO_2 _incubator. After washing with 1 x PBS^-^, the secondary antibodies conjugated with Alexa Fluor 488 or 594 (Invitrogen Life Technologies Japan, Tokyo) were applied for 1 hr in the incubator. All antibodies were diluted in TBS-DT (20 mM Tris-HCl, pH7.6, 137 mM NaCl, 0.1% Tween 20, 125 μg/ml ampicillin, 5% skim milk). After washing with 1 x PBS^-^, the coverslips were mounted onto slide glasses with 10% Glycerol in 1 x PBS^-^. Nucleus was counterstained with DAPI. The primary antibodies used in this study were mouse anti-phosphorylated histone H2AX at serine 139 monoclonal antibody (clone 2F3, BioLegend, San Diego, CA), rabbit anti-phosphorylated histone H2AX at serine 139 polyclonal antibody (A300-081A, BETHYL, Montgomery, TX), mouse anti-phosphorylated histone H3 at serine 10 monoclonal antibody (Clone 3H10, Millipore Japan, Tokyo), rabbit anti-phosphorylated histone H3 at serine 10 polyclonal antibody (06-570, Millipore Japan, Tokyo).

### Determination of Mitotic cells

Cells were incubated with anti-phosphorylated histone H3 at serine 10 followed by the incubation with the Alexa Fluor-labeled secondary antibody. The samples were scanned and imaged using IN Cell Analyzer 1000 (GE Healthcare Japan, Tokyo). Two-dimensional digital images were acquired using a 20X, 0.45NA objective lens and a 12-bit charged coupled device camera (GE Healthcare Japan, Tokyo). Images were processed and analyzed by IN Cell Investigator software (GE Healthcare Japan, Tokyo), and a fraction of cells with strong fluorescence signal was gated as mitotic cells. The original images corresponding to these cells were recalled and nuclear morphology was examined. Cells with condensed chromosomes were judged as mitotic cells. More than 5000 cells were analyzed per point.

### Measurement of the SOID of phosphorylated-H2AX foci

The samples were scanned and imaged using IN Cell Analyzer 1000 (GE Healthcare Japan, Tokyo). Two-dimensional digital images were acquired using a 20X, 0.45NA objective lens and a 12-bit charged coupled device camera (GE Healthcare Japan, Tokyo). All images were captured with the same condition so that the background intensities were almost the same throughout the same series of experiments. Images were processed and analyzed by IN Cell Investigator software (GE Healthcare Japan, Tokyo). Nuclear area was determined by the DAPI fluorescence signal. Area (total pixel number) and mean fluorescence intensity per pixel of each phosphorylated-H2AX focus, and the number of foci per cell were obtained by IN Cell Investigator software using the original parameters provided by IN Cell Developer software (GE Healthcare Japan, Tokyo). Then, the SOID was calculated by IN Cell Investigator.

The SOID was defined as the sum of fluorescence of each focus within one nucleus. The SOID was calculated for individual nucleus as the sum of (area (total pixel numbers) of each focus) x (mean fluorescence intensity per pixel of each focus). We set background threshold of foci so that the foci number scored by IN Cell Analyzer is identical to that scored by eye. To compare the SOID values between G2 and mitotic cells, G2 cells were discriminated from mitotic cells based upon nuclear morphology and phosphorylated histone H3 signal. G2 cells had weaker intensity and more rugged and discontinuous pattern of phosphorylated histone H3 signal compared to mitotic cells. Mitotic cells showed condensed chromosomes, which became visible in prophase. As phosphorylated histone H2AX foci in metaphases and anaphases were not suitable for proper quantitative analysis, the SOID value in mitotic cells was calculated predominantly in prophases. Approximately 5000 cells from multiple coverslips were scanned.

### Preparation of chromosome samples and chromosome analysis

Exponentially-growing normal human primary fibroblasts were treated with 10 μM KU55933, or its solvent DMSO 30 min before irradiation. Then, cells were irradiated with 0.02, 0.04, 0.08, 0.1, 0.2, and 0.4 Gy of X-rays. Immediately after irradiation, Colcemid (Invitrogen Life Technologies Japan, Tokyo) was added at a final concentration of 0.1 μg/ml to collect metaphase cells. Two hours later, metaphases were harvested by brief trypsinization and tapping flasks. KU55933 was washed out 30 min before metaphase harvest to make experimental condition identical to the SOID experiment. Harvested cells were once washed with 1 x PBS^-^, and then, 0.075 M KCl was treated for 20 min at ambient temperature to swell cells. After the hypotonic treatment, cells were fixed with Carnoy's fixative (methanol : acetic acid = 3 : 1) for 30 min on ice. Then, cells were resuspended with appropriate volume of Carnoy's fixative, and dropped onto 70% ethanol-immersed slide glasses. After drying overnight, slide glasses were stained with 6.5% Giemsa staining solution. Chromatid breaks were scored by eye, and at least 50 metaphases were analyzed per point.

### Data analysis

Wilcoxon rank test was used to evaluate significant difference between two groups. *P *values of less than 0.05 were considered significant difference.

## Competing interests

The authors declare that they have no competing interests.

## Authors' contributions

AI conceived of the study, carried out the immunoflorescence study, and drafted the manuscript. MY carried out the immunoflorescence study, performed the statistical analysis, and drafted the manuscript. KS participated in the design of the study. SY helped to draft the manuscript. All authors read and approved the final manuscript.
